# Polycystic Liver Disease and Sarcoidosis: Unusual Coexisting Etiologies of Portal Hypertension

**DOI:** 10.7759/cureus.996

**Published:** 2017-01-25

**Authors:** Waseem Amjad, Sophia Jagroop, Rukma Parthvi

**Affiliations:** 1 Forest Hills Hospital, Northshore-Long Island Jewish Health System

**Keywords:** sarcoidosis, polycystic liver disease, portal hypertesnion, cirrhosis

## Abstract

Both polycystic liver disease (PLD) and sarcoidosis can involve liver. Most of the time, liver disease in both conditions is asymptomatic, but they can rarely cause portal hypertension. Our aim is to report a case of a 51-year-old female with a history of adult dominant polycystic kidney disease (ADPKD) and sarcoidosis who presented with multiple episodes of hematemesis. An endoscopy showed grade 3 esophageal varices. A computed tomography (CT) scan of the abdomen showed ascites with polycystic liver, nodular contour, and calcified granuloma. PLD can cause portal hypertension due to fibrosis or large cysts compressing on the portal vein. On the other hand, sarcoidosis causes portal hypertension by formation of arteriovenous(AV) shunts or fibrosis in areas of granulomas. Both conditions are diagnosed on imaging. There is no approved medical treatment for PLD; the only curative treatment is liver transplantation. Asymptomatic hepatic sarcoidosis does not need any treatment. The recommended treatment is corticosteroids for both isolated and systemic sarcoidosis. ADPKD and sarcoidosis can involve multiple organs. The presence of both conditions can accelerate the disease process and could be a therapeutic challenge. Early abdominal imaging during the course of both diseases can improve the outcome by decreasing the diagnostic window.

## Introduction

Polycystic liver disease (PLD) is one of the rare conditions with a prevalence of 1/100,000 to 1/1000,000. Although typically asymptomatic, PLD can complicate the hemorrhage due to rupture of cysts, infection, and portal hypertension and, rarely, end stage liver disease [1]. There are very few cases in literature where polycystic liver disease presented with complication of portal hypertension. Sarcoidosis is a systemic granulomatous disease; it can involve the liver, but in most of the cases patients are asymptomatic. Cirrhosis and portal hypertension develop in less than one percent cases of sarcoidosis [2]. We present a case where adult dominant polycystic kidney disease (ADPKD) and sarcoidosis coexisted and presented as decompensated liver disease.

## Case presentation

**Figure 1:**
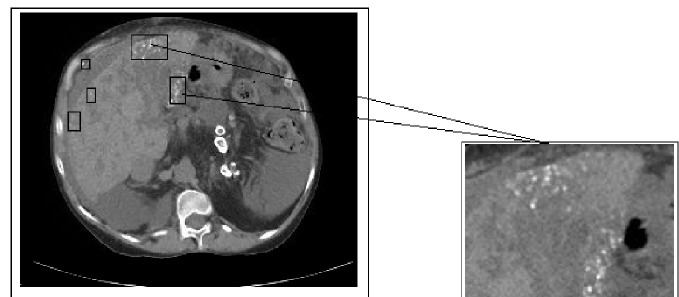
Abdominal CT scan showing multiple cystic hypo-densities with calcification in liver parenchyma.

A 51-year-old female presented to the emergency department after several episodes of hematemesis and severe epigastric pain. She had a past medical history of pulmonary sarcoidosis, ADPKD requiring bilateral nephrectomy and left kidney transplant, and is currently on immunosuppressive therapy. On admission, her vitals were stable. Her hemoglobin was 9.8 g/dl. Her total bilirubin was 0.2 mg/dl, alkaline phosphates 74 U/L, aspartate aminotransferase 24 U/L, alanine aminotransferase 20 U/L, blood urea nitrogen 49 mg/dl, and creatinine 1.07 mg/dl. Electrolytes were normal. She underwent emergent esophagogastroduodenoscopy (EGD), which showed grade 3 esophageal varices and four of them were banded. Octreotide and pantoprazole drip were administered. A computed tomography (CT) scan of the abdomen showed ascites with polycystic liver, nodular contour, and several sites of calcifications (most likely calcified granulomas) (Figure 1). An abdominal ultrasound showed a heterogeneous liver with mild contour and patent portal circulation. A previous magnetic resonance imaging (MRI) of the pelvis, which was done two years ago, showed similar small cysts in the parenchyma; however, the calcifications and nodularity of the liver were new. Diagnostic paracentesis revealed serum to ascitic albumin gradient (SAAG) of 1.9 consistent with portal hypertension. The patient was started on non-selective beta blocker for stabilization of varices. Ceftriaxone was given for prophylaxis for spontaneous bacterial peritonitis. The patient’s bleeding was controlled, and the hemoglobin level was stable during the hospital stay. She refused further workup including liver biopsy. 

## Discussion

Polycystic liver disease occurs either in isolation or as part of ADPKD or uncommonly adult dominant polycystic liver disease (ADPLD). Polycystic liver disease involves mutation of polycystin 1, polycystin 2, hepatocystin, and Sec63, which leads to the failure of intralobular bile ducts to involute during embryonic development. The risk factors for hepatic cyst growth involve age, female gender, and size of renal cysts [3]. Hepatic cystic disease is usually asymptomatic and does not need intervention; rarely, extensive cystic disease may lead to liver failure. There are few cases in literature where polycystic liver disease complicated into portal hypertension either with fibrosis or with extensive cystic disease [4-5]. The pathogenesis includes two processes. First, polycystic disease decreases hepatic vein flow, and second cysts can compress the portal vein leading to portal hypertension [1].

Sarcoidosis is a systemic granulomatous disease that can commonly involve the liver. Hepatic sarcoidosis is usually asymptomatic, and rarely can it progress to cirrhosis, portal hypertension, cholestasis, and Budd-Chiari syndrome [6]. The pathophysiology of portal hypertension in sarcoidosis is unknown. Formation of AV shunts in areas of granulomas could be a cause; AV shunting increases the resistance which leads to portal hypertension. Another suggestion is that healing fibrosis in areas of granuloma increases the resistance, which can lead to portal hypertension with cirrhosis. Another theory suggests that ischemic changes caused by granulomatous phlebitis of portal and hepatic veins can lead to cirrhosis and portal hypertension [2].

Both conditions are diagnosed on imaging. Abdominal ultrasound and CT scan are the first line modalities. Liver biopsy supports diagnosis of sarcoidosis and rules out other conditions. Liver function abnormalities are common in symptomatic patients [3,6]. The presence of both sarcoidosis and polycystic liver disease increases the risk of development of decompensated liver disease or liver failure as compared to single disease. To our knowledge there is no case in literature where both conditions involved the liver simultaneously.

There is no approved medical treatment for PLD. Studies have shown somatostatin analogues and mammalian target of rapamycin (mTOR) inhibitors efficacy as compared to placebo. Radiological treatment includes aspiration followed by sclerotherapy for large cysts. Surgical treatment involves fenestration, segmental hepatic resection, and liver transplantation. Liver transplantation is the only curative treatment for PLD [3]. The Model of End Stage Liver Disease (MELD) score is commonly used for liver allocation. Usually liver functions are preserved in PLD, and the MELD score is not the best criteria to assess for liver transplantation. Some studies suggested the use of the MELD priority score to improve the outcome [7]. Asymptomatic hepatic sarcoidosis patients do not need any treatment. The recommended treatment is corticosteroid for both isolated and systemic sarcoidosis. Corticosteroids improve liver function tests but do not improve progression of disease. The survival in patients who have undergone a liver transplant with hepatic sarcoidosis is comparable to other liver diseases leading to transplant [8].

## Conclusions

Polycystic liver disease and sarcoidosis are rare etiologies for portal hypertension. The presence of both conditions can accelerate the disease process and could be a therapeutic challenge. An early abdominal imaging during the course of a disease can improve the outcome.
